# Supplementary material for: Continuous Piperacillin-Tazobactam Infusion Improves Clinical Outcomes in Critically Ill Patients with Sepsis: A Retrospective, Single-Centre Study
Source: Antibiotics (Basel). 2022 Oct 29;11(11):1508. doi: 10.3390/antibiotics11111508 (PMC9686508; doi:10.3390/antibiotics11111508)
Supplement: Supplementary file 1 [file antibiotics-11-01508-s001.zip › antibiotics-1965385-supplementary.pdf]

Supplemental Table S1. Cox proportional hazards model of factors associated with mortality at 28 days

	Univariable analysis			Multivariable analysis		
**Variable**	**OR**	**95% CI**	***p*-value**	**OR**	**95% CI**	***p*-value**
Age						
≥ 68 years	0.751	0.390–1.444	0.390			
Female (versus male)	1.087	0.535–2.211	0.817			
BMI ≥ 23 kg/m^2	0.831	0.431–1.604	0.582			
Co-existing condition						
Congestive heart failure	2.376	0.923–6.120	0.073			
Respiratory failure	0.560	0.198–1.583	0.274			
Chronic liver disease	2.306	0.814–6.531	0.116			
End-stage renal disease	1.151	0.406–3.257	0.792			
Immunocompromised	0.671	0.237–1.899	0.452			
Organ dysfunction						
Cardiovascular	2.280	1.039–5.004	0.040	2.285	1.008-5.180	0.048
Respiratory	0.410	0.097–1.726	0.224			
Renal	1.205	0.616–2.356	0.586			
Haematological	1.721	0.892–3.323	0.106			
Severity of illness						
SOFA ≥ 9	2.165	1.043–4.491	0.016			
APACHE II ≥ 18	1.498	0.758–2.958	0.245			
Septic shock	2.671	1.388–5.140	0.003			
Primary site of infection						
Pulmonary site	0.716	0.253–2.029	0.530			
Urinary site	35.164	3.572–346.144	0.002	36.584	3.627-368.977	0.002
Blood	4.268	1.496–12.172	0.007			
Other site	1.544	0.211–11.296	0.669			
Unknown	1.544	0.473–5.043	0.472			
Parameters of piperacillin-tazobactam						
Continuous infusion	0.456	0.190–1.095	0.079	0.309	0.123-0.777	0.013
Concomitant antibiotic						
Teicoplanin	0.854	0.401–1.817	0.682			
Levofloxacin	0.932	0.459–1.894	0.846			
TMP-SMX	1.730	0.788–3.797	0.172			
Other antibiotics	0.638	0.196–2.082	0.457			
Laboratory variables						
Lactate ≥ 2 mmol/L	2.162	1.106–4.228	0.024	1.936	0.949-4.603	0.070
CRP ≥ 10 mg/dL	1.201	0.619–2.331	0.589			
WBC ≥ 11 × 10^3/L	1.102	0.571–2.126	0.773			

Pre-enrollment status						
ICU LOS ≥ 1 day	0.965	0.440–2.118	0.929			
Hospital LOS ≥ 1 day	1.033	0.535–1.994	0.924			
Number of antibiotics ≥ 1	1.278	0.664–2.457	0.463	2.623	1.113-4.603	0.024
Organ support at baseline						
Mechanical ventilation	1.723	0.717–4.140	0.224			
RRT	2.153	1.012–4.583	0.047	2.198	1.005-4.809	0.049
ECMO	0.704	0.096–5.143	0.730			
Positive blood culture	2.425	1.061–5.542	0.036			
Gram-positive	0.212	0.042–1.075	0.061			
Gram-negative	5.306	0.920–30.604	0.062			
Susceptible to drug	3.359	0.732–15.414	0.119			
Positive sputum culture	1.052	0.538–2.056	0.883			
Gram-positive	0.733	0.164–3.281	0.685			
Gram-negative	1.413	0.495–4.029	0.518			
Multidrug-resistant GNB	2.241	0.624–8.049	0.216			
Pneumocystis jirovecii	1.245	0.347–4.471	0.736			
Others	0.837	0.262–2.668	0.763			
Susceptible to drug	0.981	0.307–3.129	0.974			
Other culture	1.700	0.408–7.081	0.466			
Gram-positive	2.828	0.170–47.146	0.469			
Gram-negative	0.354	0.021–5.893	0.469			

Abbreviations: OR, odds ratio; CI, confidence interval; BMI, body mass index; SOFA, Sequential Organ Failure Assessment score; APACHE, Acute Physiology and Chronic Health Evaluation; TMP-SMX, trimethoprim sulfamethoxazole; ICU, intensive care unit; LOS, length of stay; CRP, C-reactive protein; WBC, white blood cell; RRT, renal replacement therapy; ECMO, extracorporeal membrane oxygenation; MRSA, methicillin-resistant Staphylococcus aureus; GNB, Gram-negative bacilli.

Supplemental Table S2. Cox proportional hazards model for factors associated with freedom from mechanical ventilation at 14 days

	Univariable analysis			Multivariable analysis		
**Variable**	**OR**	**95% CI**	***p*-value**	**OR**	**95% CI**	***p*-value**
Age						
≥ 68 years	0.857	0.549-1.339	0.498			
Female (versus male)	1.223	0.760-1.967	0.406			
BMI ≥ 23 kg/m^2	0.891	0.572-1.388	0.611			
Co-existing condition						
Congestive heart failure	2.010	0.807-5.007	0.134			
Respiratory failure	1.122	0.656-1.921	0.674			
Chronic liver disease	1.710	0.538-5.432	0.363			
End-stage renal disease	1.651	0.849-3.211	0.139			
Immunocompromised	0.707	0.364-1.374	0.307			
Organ dysfunction						
Cardiovascular	0.683	0.429-1.086	0.107			
Respiratory	0.224	0.078-0.643	0.005			
Renal	0.757	0.477-1.200	0.236			
Haematological	0.899	0.569-1.422	0.650			
Severity of illness						
SOFA ≥ 9	0.565	0.357-0.894	0.015	0.638	0.401-1.015	0.058
APACHE II ≥ 18	0.575	0.366-0.903	0.016			
Septic shock	0.895	0.564-1.420	0.639			
Primary site of infection						
Pulmonary site	0.779	0.357-1.702	0.531			
Abdominal	1.903	0.461-7.855	0.374			
Other site	0.793	0.195-3.230	0.746			
Unknown	2.023	0.812-5.038	0.130			
Parameters of piperacillin-tazobactam						
Continuous infusion	1.581	0.995-2.510	0.052	1.707	1.063-2.740	0.027
Concomitant antibiotic						
Teicoplanin	0.604	0.356-1.024	0.061			
Levofloxacin	1.004	0.621-1.623	0.986			
TMP-SMX	0.779	0.401-1.515	0.463			
Other antibiotics	1.794	0.977-3.292	0.059			
Laboratory variables						
Lactate ≥ 2 mmol/L	1.997	0.964-4.138	0.063			
CRP ≥ 10 mg/dL	0.509	0.324-0.799	0.003	0.557	0.352-0.882	0.013
WBC ≥ 11 × 10^3/L	1.016	0.646-1.596	0.947			
Pre-enrollment status						
ICU LOS ≥ 1 day	1.310	0.780-2.199	0.307			
Hospital LOS ≥ 1 day	1.311	0.841-2.044	0.232			
Number of antibiotics ≥ 1	0.931	0.595-1.456	0.753			
Organ support at baseline						
RRT	0.750	0.386-1.456	0.395			
ECMO	3.052	1.097-8.486	0.033			
Positive blood culture	0.357	0.130-0.978	0.045	0.430	0.154-1.204	0.108
Positive sputum culture	0.794	0.506-1.246	0.315			

Other culture	0.632	0.155-2.576	0.522

Abbreviations: OR, odds ratio; CI, confidence interval; BMI, body mass index; SOFA, Sequential Organ Failure Assessment score; APACHE, Acute Physiology and Chronic Health Evaluation; TMP-SMX, trimethoprim sulfamethoxazole; ICU, intensive care unit; LOS, length of stay; CRP, C-reactive protein; WBC, white blood cell; RRT, renal replacement therapy; ECMO, extracorporeal membrane oxygenation.

Supplemental Table S3. Cox proportional hazards model for factors associated with freedom from mechanical ventilation at 28 days

	Univariable analysis			Multivariable analysis		
**Variable**	**OR**	**95% CI**	***p*-value**	**OR**	**95% CI**	***p*-value**
Age						
≥ 68 years	0.929	0.587-1.469	0.752			
Female (versus male)	1.331	0.828-2.142	0.238			
BMI ≥ 23 kg/m^2	0.743	0.471-1.173	0.203			
Co-existing condition						
Congestive heart failure	3.940	1.370-11.331	0.011	3.193	1.055-9.663	0.394
Respiratory failure	1.293	0.768-2.177	0.334			
Chronic liver disease	5.080	1.202-21.473	0.027			
End-stage renal disease	1.447	0.742-2.825	0.279			
Immunocompromised	0.493	0.236-1.028	0.059			
Organ dysfunction						
Cardiovascular	0.740	0.459-1.194	0.217			
Respiratory	0.292	0.089-0.961	0.043	0.616	0.164-2.318	0.474
Renal	0.746	0.466-1.196	0.224			
Haematological	0.831	0.515-1.341	0.448			
Severity of illness						
SOFA ≥ 9	0.577	0.362-0.920	0.021	0.907	0.525-1.566	0.907
APACHE II ≥ 18	0.541	0.339-0.863	0.010	0.768	0.434-1.360	0.366
Septic shock	0.833	0.516-1.345	0.455			
Primary site of infection						
Pulmonary site	0.643	0.292-1.419	0.274			
Abdominal	1.676	0.406-6.923	0.475			
Other site	0.529	0.073-3.812	0.527			
Unknown	4.558	1.732-11.991	0.002	2.942	1.000-8.655	0.050
Parameters of piperacillin-tazobactam						
Continuous infusion	1.323	0.830-2.107	0.239	1.253	0.761-2.062	0.376
Concomitant antibiotic						
Teicoplanin	0.612	0.362-1.033	0.066			
Levofloxacin	0.934	0.572-1.525	0.784			
TMP-SMX	0.793	0.364-1.726	0.558			
Other antibiotics	1.397	0.746-2.617	0.297			
Laboratory variables						
Lactate ≥ 2 mmol/L	0.931	0.591-1.466	0.757			
CRP ≥ 10 mg/dL	0.443	0.277-0.710	0.001	0.504	0.305-0.833	0.008
WBC ≥ 11 × 10^3/L	1.000	0.632-1.582	1.000			
Pre-enrollment status						
ICU LOS ≥ 1 day	1.291	0.767-2.173	0.337			
Hospital LOS ≥ 1 day	1.326	0.844-2.084	0.221			
Number of antibiotics ≥ 1	0.967	0.612-1.529	0.887			
Organ support at baseline						
RRT	0.804	0.412-1.569	0.523			
ECMO	2.665	0.958-7.411	0.060			
Positive blood culture	0.284	0.089-0.906	0.033	0.362	0.110-1.191	0.095
Positive sputum culture	0.604	0.377-0.967	0.036	0.674	0.402-1.129	0.134

Other culture	0.502	0.123-2.049	0.337

Abbreviations: OR, odds ratio; CI, confidence interval; BMI, body mass index; SOFA, Sequential Organ Failure Assessment score; APACHE, Acute Physiology and Chronic Health Evaluation; TMP-SMX, trimethoprim sulfamethoxazole; ICU, intensive care unit; LOS, length of stay; CRP, C-reactive protein; WBC, white blood cell; RRT, renal replacement therapy; ECMO, extracorporeal membrane oxygenation.

Supplemental Table S4. Cox proportional hazards model for factors associated with discharge alive from the ICU at 14 days

	Univariable analysis			Multivariable analysis		
**Variable**	**OR**	**95% CI**	***p*-value**	**OR**	**95% CI**	***p*-value**
Age						
≥ 68 years	0.923	0.617-1.380	0.695			
Female (versus male)	1.244	0.810-1.911	0.319			
BMI ≥ 23 kg/m^2	0.942	0.631-1.408	0.772			
Co-existing condition						
Congestive heart failure	1.675	0.773-3.628	0.191			
Respiratory failure	0.983	0.582-1.661	0.948			
Chronic liver disease	1.864	0.755-4.600	0.177			
End-stage renal disease	1.384	0.738-2.595	0.311			
Immunocompromised	0.898	0.509-1.583	0.709			
Organ dysfunction						
Cardiovascular	0.603	0.403-0.902	0.014	0.689	0.440-1.080	0.104
Respiratory	0.278	0.110-0.699	0.007	0.521	0.192-1.412	0.200
Renal	0.917	0.601-1.398	0.687			
Haematological	0.995	0.663-1.493	0.980			
Severity of illness						
SOFA ≥ 9	0.575	0.385-0.861	0.007			
APACHE II ≥ 18	1.714	1.145-2.565	0.009			
Septic shock	0.819	0.522-1.284	0.384			
Primary site of infection						
Pulmonary site	0.568	0.315-1.026	0.061	0.792	0.403-1.557	0.499
Abdominal	4.233	1.311-13.663	0.016	4.456	1.101-18.031	0.036
Urinary site	3.526	0.483-25.737	0.214			
Skin or soft tissue	1.583	0.220-11.410	0.649			
Central nervous system	1.285	0.179-9.250	0.803			
Other site	1.147	0.363-3.625	0.816			
Unknown	1.213	0.560-2.625	0.625			
Parameters of piperacillin-tazobactam						
Continuous infusion	1.676	1.107-2.539	0.015	1.938	1.269-3.007	0.002
Concomitant antibiotic						
Teicoplanin	0.604	0.369-0.990	0.045	0.604	0.361-1.011	0.055
Levofloxacin	1.048	0.675-1.627	0.835			
TMP-SMX	0.559	0.290-1.079	0.083	0.622	0.313-1.233	0.174
Other antibiotics	1.456	0.848-2.500	0.174			
Laboratory variables						
Lactate ≥ 2 mmol/L	0.934	0.622-1.401	0.741			
CRP ≥ 10 mg/dL	0.568	0.377-0.854	0.007	0.654	0.423-1.011	0.056
WBC ≥ 11 × 10^3/L	0.934	0.624-1.396	0.738			
Pre-enrollment status						
ICU LOS ≥ 1 day	1.438	0.911-2.270	0.118			
Hospital LOS ≥ 1 day	1.224	0.817-1.832	0.327			
Number of antibiotics ≥ 1	1.178	0.788-1.760	0.425			
Organ support at baseline						
Mechanical ventilation	0.683	0.440-1.062	0.091	0.727	0.449-1.179	0.196

RRT	0.736	0.392-1.379	0.338
ECMO	1.974	0.795-4.898	0.143
Positive blood culture	0.719	0.333-1.555	0.403
Positive sputum culture	0.805	0.529-1.224	0.311
Other culture	0.610	0.150-2.478	0.490

Abbreviations: OR, odds ratio; CI, confidence interval; BMI, body mass index; SOFA, Sequential Organ Failure Assessment score; APACHE, Acute Physiology and Chronic Health Evaluation; TMP-SMX, trimethoprim sulfamethoxazole; ICU, intensive care unit; LOS, length of stay; CRP, C-reactive protein; WBC, white blood cell; RRT, renal replacement therapy; ECMO, extracorporeal membrane oxygenation.

Supplemental Table S5. Cox proportional hazards model for factors associated with discharge alive from the ICU at 28 days

	Univariable analysis			Multivariable analysis		
**Variable**	**OR**	**95% CI**	***p*-value**	**OR**	**95% CI**	***p*-value**
Age						
≥ 68 years	0.963	0.655-1.414	0.846			
Female (versus male)	1.387	0.922-2.086	0.116			
BMI ≥ 23 kg/m^2	0.730	0.496-1.074	0.111			
Co-existing condition						
Congestive heart failure	1.521	0.664-3.483	0.321			
Respiratory failure	0.893	0.543-1.470	0.657			
Chronic liver disease	2.971	1.185-7.447	0.020	2.724	1.009-7.350	0.048
End-stage renal disease	1.385	0.789-2.433	0.257			
Immunocompromised	0.730	0.422-1.262	0.260			
Organ dysfunction						
Cardiovascular	0.646	0.438-0.951	0.027	0.779	0.479-1.266	0.313
Respiratory	0.358	0.129-0.992	0.048	0.720	0.238-2.172	0.559
Renal	0.870	0.582-1.302	0.500			
Haematological	0.978	0.661-1.447	0.912			
Severity of illness						
SOFA ≥ 9	0.655	0.446-0.962	0.031	0.880	0.515-1.501	0.638
APACHE II ≥ 18	0.617	0.420-0.908	0.014	0.836	0.504-1.384	0.486
Septic shock	0.818	0.531-1.260	0.363			
Primary site of infection						
Pulmonary site	0.547	0.301-0.994	0.048	0.716	0.361-1.418	0.337
Abdominal	3.834	1.187-12.383	0.025	5.071	1.277-20.144	0.021
Urinary site	3.198	0.438-23.365	0.252			
Skin or soft tissue	1.375	0.191-9.917	0.752			
Central nervous system	1.098	0.152-7.902	0.926			
Other site	1.123	0.276-4.566	0.871			
Unknown	1.832	0.835-4.020	0.131			
Parameters of piperacillin-tazobactam						
Continuous infusion	1.277	0.855-1.907	0.232	1.214	0.811-1.818	0.347
Concomitant antibiotic						
Teicoplanin	0.640	0.410-1.000	0.050	0.741	0.455-1.205	0.227
Levofloxacin	0.978	0.644-1.487	0.919			
TMP-SMX	0.676	0.361-1.267	0.222			
Other antibiotics	1.356	0.790-2.327	0.270			
Laboratory variables						
Lactate ≥ 2 mmol/L	0.901	0.611-1.327	0.597			
CRP ≥ 10 mg/dL	0.503	0.339-0.746	0.001	0.580	0.375-0.898	0.015
WBC ≥ 11 × 10^3/L	0.881	0.602-1.291	0.516			
Pre-enrollment status						
ICU LOS ≥ 1 day	1.313	0.848-2.034	0.222			
Hospital LOS ≥ 1 day	1.163	0.792-1.706	0.441			
Number of antibiotics ≥ 1	1.167	0.795-1.712	0.430			
Organ support at baseline						
Mechanical ventilation	0.737	0.480-1.131	0.162			

RRT	0.824	0.469-1.447	0.500
ECMO	1.720	0.693-4.269	0.242
Positive blood culture	0.597	0.261-1.362	0.220
Positive sputum culture	0.735	0.491-1.099	0.134
Other culture	0.647	0.205-2.042	0.458

Abbreviations: OR, odds ratio; CI, confidence interval; BMI, body mass index; SOFA, Sequential Organ Failure Assessment score; APACHE, Acute Physiology and Chronic Health Evaluation; TMP-SMX, trimethoprim sulfamethoxazole; ICU, intensive care unit; LOS, length of stay; CRP, C-reactive protein; WBC, white blood cell; RRT, renal replacement therapy; ECMO, extracorporeal membrane oxygenation.

Supplemental Table S6. Sequential Organ Failure Assessment (SOFA) score [1]

SOFA score					
Variables	0	1	2	3	4
PaO2/FiO2, mmHg	≥ 400	< 400	< 300	< 200 with respiratory support	< 100 with respiratory support
Platelets, 1000/uL	≥ 150	< 150	< 100	< 50	< 20
Bilirubin, mg/dL	< 1.2	1.2-1.9	2.0-5.9	6.0-11.9	> 12.0
Mean arterial pressure, mmHg	≥ 70mmHg	< 70	Dopamine < 5 mcg/kg/min or Dobutamine (any dose)	Dopamine 5.1-15 mcg/kg/min or epinephrine ≤ 0.1 mcg/kg/min or norepinephrine ≤ 0.1 mcg/kg/min	Dopamine > 15 mcg/kg/min or epinephrine > 0.1 mcg/kg/min or norepinephrine > 0.1 mcg/kg/min
Glasgow Coma Scale score	15	13-14	10-12	6-9	<6
Creatinine, mg/dL	<1.2	1.2-1.9	2.0-3.4	3.5-4.9	> 5.0

Abbreviations: PaO2, partial pressure of oxygen; FiO2, Fraction of inspired oxygen.

Supplemental Table S7. Acute Physiology and Chronic Health Evaluation II (APACHE II) score [2]

Physiologic variable		Point Score								
		+4	+3	+2	+1	0	+1	+2	+3	+4
1	Temperature, º	≥ 41	39-40.9		38.5-38.9	36-38.4	34-35.9	32-33.9	30-31.9	≤ 29.9
2	Mean arterial pressure, mmHg	≥ 160	130-159	110-129		70-109		50-69		≤ 49
3	Heart rate	≥ 180	140-179	110-139	25-34	70-109		50-69	40-54	≤ 39
4	Respiratory rate	≥ 50	35-49		25-34	12-24	10-11	6-9		≤ 5
5	Oxygenation:									
	a) FiO2 ≥ 0.5: A-aDO2	≥ 500	350-499	200-349		< 200				
	b) FiO2 <: PaO2					> 70	61-70		55-60	< 55
6	Arterial pH	≥ 7.7	7.6-7.69		7.5-7.59	7.33-7.49		7.25-7.32	7.15-7.24	< 7.15
7	Serum Na, mMol/L	≥ 180	160-179	155-159	150-154	130-149		120-129	111-119	≤ 110
8	Serum K, mMol/L	≥ 7	6-6.9		5.5-5.9	3.5-5.4	3-3.4	2.5-2.9		< 2.5
9	Serum creatinine, mg/dL	≥ 3.5	2-3.4	1.5-1.9		0.6-1.4		< 0.6		
10	Hematocrit, %	≥ 60		50-59.9	46-49.9	30-45.9		20-29.9		< 20
11	White blood cell, *1000	≥ 40		20-39.9	15-19.9	3-14.9		1-2.9		< 1
12	Glasgow coma scale	Score = 15 – actual Glasgow coma scale								

Abbreviations: FiO2, Fraction of inspired oxygen; A-aDO2, alveolar-arterial oxygen pressure difference; PaO2, partial pressure of oxygen.

References

1. Jones, A.E., S. Trzeciak, and J.A. Kline, The Sequential Organ Failure Assessment score for predicting outcome in patients with severe sepsis and evidence of hypoperfusion at the time of emergency department presentation. Crit Care Med. 2009;37(5):1649-54.
2. Knaus W.A., Draper E.A., Wagner D.P., Zimmerman J.E., APACHE II: a severity of disease classification system. Crit Care Med. 1985;13(10):818-29.
